# Supplementary material for: COZOID: contact zone identifier for visual analysis of protein-protein interactions
Source: BMC Bioinformatics. 2018 Apr 6;19:125. doi: 10.1186/s12859-018-2113-6 (PMC5889581; doi:10.1186/s12859-018-2113-6)
Supplement: Supplementary file 2 — Software build. Executable binary file of the software tool. (ZIP 118,784 kb) [file 12859_2018_2113_MOESM2_ESM.zip › cozoid/java/docs/beansbinding-1.2.1-doc.zip!/org/jdesktop/el/BeanELResolver.html]

BeanELResolver (Beans Binding)


---


|  |  |  |
| --- | --- | --- |
| **Overview** \| **Package** \| **Class** \| **Use** \| **Tree** \| **Deprecated** \| **Index** \| **Help** | |  |
| **PREV CLASS**   **NEXT CLASS** | **FRAMES**    **NO FRAMES**     **All Classes** |  |
| SUMMARY: NESTED \| FIELD \| CONSTR \| METHOD | DETAIL: FIELD \| CONSTR \| METHOD |  |


---


## org.jdesktop.el Class BeanELResolver

```
java.lang.Object
  org.jdesktop.el.ELResolver
      org.jdesktop.el.BeanELResolver
```

---

``` public class BeanELResolver extends ELResolver ```

Defines property resolution behavior on objects using the JavaBeans
component architecture.

This resolver handles base objects of any type, as long as the
base is not `null`. It accepts any object as a property, and
coerces it to a string. That string is then used to find a JavaBeans
compliant property on the base object. The value is accessed using
JavaBeans getters and setters.

This resolver can be constructed in read-only mode, which means that
`isReadOnly` will always return `true` and
`setValue(org.jdesktop.el.ELContext, java.lang.Object, java.lang.Object, java.lang.Object)` will always throw
`PropertyNotWritableException`.

`ELResolver`s are combined together using
`CompositeELResolver`s, to define rich semantics for evaluating
an expression. See the javadocs for `ELResolver` for details.

Because this resolver handles base objects of any type, it should
be placed near the end of a composite resolver. Otherwise, it will
claim to have resolved a property before any resolvers that come after
it get a chance to test if they can do so as well.

**Since:**
:   JSP 2.1

**See Also:**: `CompositeELResolver`, `ELResolver`

---

| **Nested Class Summary** | |
| --- | --- |
| `protected static class` | `BeanELResolver.BeanProperties` |
| `protected static class` | `BeanELResolver.BeanProperty` |


**Field Summary**

**Fields inherited from class org.jdesktop.el.ELResolver**

`RESOLVABLE_AT_DESIGN_TIME, TYPE`


**Constructor Summary**

`BeanELResolver()`
  
          Creates a new read/write `BeanELResolver`.

`BeanELResolver(boolean isReadOnly)`
  
          Creates a new `BeanELResolver` whose read-only status is
determined by the given parameter.


| **Method Summary** | |
| --- | --- |
| `java.lang.Class<?>` | `getCommonPropertyType(ELContext context, java.lang.Object base)`             If the base object is not `null`, returns the most general type that this resolver accepts for the `property` argument. |
| `java.util.Iterator<java.beans.FeatureDescriptor>` | `getFeatureDescriptors(ELContext context, java.lang.Object base)`             If the base object is not `null`, returns an `Iterator` containing the set of JavaBeans properties available on the given object. |
| `java.lang.Class<?>` | `getType(ELContext context, java.lang.Object base, java.lang.Object property)`             If the base object is not `null`, returns the most general acceptable type that can be set on this bean property. |
| `java.lang.Object` | `getValue(ELContext context, java.lang.Object base, java.lang.Object property)`             If the base object is not `null`, returns the current value of the given property on this bean. |
| `boolean` | `isReadOnly(ELContext context, java.lang.Object base, java.lang.Object property)`             If the base object is not `null`, returns whether a call to `setValue(org.jdesktop.el.ELContext, java.lang.Object, java.lang.Object, java.lang.Object)` will always fail. |
| `void` | `setValue(ELContext context, java.lang.Object base, java.lang.Object property, java.lang.Object val)`             If the base object is not `null`, attempts to set the value of the given property on this bean. |

**Methods inherited from class java.lang.Object**

`clone, equals, finalize, getClass, hashCode, notify, notifyAll, toString, wait, wait, wait`

**Constructor Detail**

### BeanELResolver

```
public BeanELResolver()
```

:   Creates a new read/write `BeanELResolver`.

---


### BeanELResolver

```
public BeanELResolver(boolean isReadOnly)
```

:   Creates a new `BeanELResolver` whose read-only status is
    determined by the given parameter.

    **Parameters:**: `isReadOnly` - `true` if this resolver cannot modify beans; `false` otherwise.


**Method Detail**

### getType

```
public java.lang.Class<?> getType(ELContext context,
                                  java.lang.Object base,
                                  java.lang.Object property)
```

:   If the base object is not `null`, returns the most
    general acceptable type that can be set on this bean property.

    If the base is not `null`, the
    `propertyResolved` property of the `ELContext`
    object must be set to `true` by this resolver, before
    returning. If this property is not `true` after this
    method is called, the caller should ignore the return value.

    The provided property will first be coerced to a `String`.
    If there is a `BeanInfoProperty` for this property and
    there were no errors retrieving it, the `propertyType` of
    the `propertyDescriptor` is returned. Otherwise, a
    `PropertyNotFoundException` is thrown.

    :   **Specified by:**: `getType` in class `ELResolver`
    :   **Parameters:**: `context` - The context of this evaluation.: `base` - The bean to analyze.: `property` - The name of the property to analyze. Will be coerced to a `String`. **Returns:**: If the `propertyResolved` property of `ELContext` was set to `true`, then the most general acceptable type; otherwise undefined. **Throws:**: `java.lang.NullPointerException` - if context is `null`: `PropertyNotFoundException` - if `base` is not `null` and the specified property does not exist or is not readable.: `ELException` - if an exception was thrown while performing the property or variable resolution. The thrown exception must be included as the cause property of this exception, if available.

---


### getValue

```
public java.lang.Object getValue(ELContext context,
                                 java.lang.Object base,
                                 java.lang.Object property)
```

:   If the base object is not `null`, returns the current
    value of the given property on this bean.

    If the base is not `null`, the
    `propertyResolved` property of the `ELContext`
    object must be set to `true` by this resolver, before
    returning. If this property is not `true` after this
    method is called, the caller should ignore the return value.

    The provided property name will first be coerced to a
    `String`. If the property is a readable property of the
    base object, as per the JavaBeans specification, then return the
    result of the getter call. If the getter throws an exception,
    it is propagated to the caller. If the property is not found or is
    not readable, a `PropertyNotFoundException` is thrown.

    :   **Specified by:**: `getValue` in class `ELResolver`
    :   **Parameters:**: `context` - The context of this evaluation.: `base` - The bean on which to get the property.: `property` - The name of the property to get. Will be coerced to a `String`. **Returns:**: If the `propertyResolved` property of `ELContext` was set to `true`, then the value of the given property. Otherwise, undefined. **Throws:**: `java.lang.NullPointerException` - if context is `null`.: `PropertyNotFoundException` - if `base` is not `null` and the specified property does not exist or is not readable.: `ELException` - if an exception was thrown while performing the property or variable resolution. The thrown exception must be included as the cause property of this exception, if available.

---


### setValue

```
public void setValue(ELContext context,
                     java.lang.Object base,
                     java.lang.Object property,
                     java.lang.Object val)
```

:   If the base object is not `null`, attempts to set the
    value of the given property on this bean.

    If the base is not `null`, the
    `propertyResolved` property of the `ELContext`
    object must be set to `true` by this resolver, before
    returning. If this property is not `true` after this
    method is called, the caller can safely assume no value was set.

    If this resolver was constructed in read-only mode, this method will
    always throw `PropertyNotWritableException`.

    The provided property name will first be coerced to a
    `String`. If property is a writable property of
    `base` (as per the JavaBeans Specification), the setter
    method is called (passing `value`). If the property exists
    but does not have a setter, then a
    `PropertyNotFoundException` is thrown. If the property
    does not exist, a `PropertyNotFoundException` is thrown.

    :   **Specified by:**: `setValue` in class `ELResolver`
    :   **Parameters:**: `context` - The context of this evaluation.: `base` - The bean on which to set the property.: `property` - The name of the property to set. Will be coerced to a `String`.: `val` - The value to be associated with the specified key. **Throws:**: `java.lang.NullPointerException` - if context is `null`.: `PropertyNotFoundException` - if `base` is not `null` and the specified property does not exist.: `PropertyNotWritableException` - if this resolver was constructed in read-only mode, or if there is no setter for the property.: `ELException` - if an exception was thrown while performing the property or variable resolution. The thrown exception must be included as the cause property of this exception, if available.

---


### isReadOnly

```
public boolean isReadOnly(ELContext context,
                          java.lang.Object base,
                          java.lang.Object property)
```

:   If the base object is not `null`, returns whether a call
    to `setValue(org.jdesktop.el.ELContext, java.lang.Object, java.lang.Object, java.lang.Object)` will always fail.

    If the base is not `null`, the
    `propertyResolved` property of the `ELContext`
    object must be set to `true` by this resolver, before
    returning. If this property is not `true` after this
    method is called, the caller can safely assume no value was set.

    If this resolver was constructed in read-only mode, this method will
    always return `true`.

    The provided property name will first be coerced to a
    `String`. If property is a writable property of
    `base`, `false` is returned. If the property is
    found but is not writable, `true` is returned. If the
    property is not found, a `PropertyNotFoundException`
    is thrown.

    :   **Specified by:**: `isReadOnly` in class `ELResolver`
    :   **Parameters:**: `context` - The context of this evaluation.: `base` - The bean to analyze.: `property` - The name of the property to analyzed. Will be coerced to a `String`. **Returns:**: If the `propertyResolved` property of `ELContext` was set to `true`, then `true` if calling the `setValue` method will always fail or `false` if it is possible that such a call may succeed; otherwise undefined. **Throws:**: `java.lang.NullPointerException` - if context is `null`: `PropertyNotFoundException` - if `base` is not `null` and the specified property does not exist.: `ELException` - if an exception was thrown while performing the property or variable resolution. The thrown exception must be included as the cause property of this exception, if available.

---


### getFeatureDescriptors

```
public java.util.Iterator<java.beans.FeatureDescriptor> getFeatureDescriptors(ELContext context,
                                                                              java.lang.Object base)
```

:   If the base object is not `null`, returns an
    `Iterator` containing the set of JavaBeans properties
    available on the given object. Otherwise, returns `null`.

    The `Iterator` returned must contain zero or more
    instances of `FeatureDescriptor`. Each info object
    contains information about a property in the bean, as obtained by
    calling the `BeanInfo.getPropertyDescriptors` method.
    The `FeatureDescriptor` is initialized using the same
    fields as are present in the `PropertyDescriptor`,
    with the additional required named attributes "`type`" and
    "`resolvableAtDesignTime`" set as follows:

    - `ELResolver.TYPE` - The runtime type of the property, from
      `PropertyDescriptor.getPropertyType()`.
    - `ELResolver.RESOLVABLE_AT_DESIGN_TIME` - `true`.

    :   **Specified by:**: `getFeatureDescriptors` in class `ELResolver`
    :   **Parameters:**: `context` - The context of this evaluation.: `base` - The bean to analyze. **Returns:**: An `Iterator` containing zero or more `FeatureDescriptor` objects, each representing a property on this bean, or `null` if the `base` object is `null`. **See Also:**: `FeatureDescriptor`

---


### getCommonPropertyType

```
public java.lang.Class<?> getCommonPropertyType(ELContext context,
                                                java.lang.Object base)
```

:   If the base object is not `null`, returns the most
    general type that this resolver accepts for the
    `property` argument. Otherwise, returns `null`.

    Assuming the base is not `null`, this method will always
    return `Object.class`. This is because any object is
    accepted as a key and is coerced into a string.

    :   **Specified by:**: `getCommonPropertyType` in class `ELResolver`
    :   **Parameters:**: `context` - The context of this evaluation.: `base` - The bean to analyze. **Returns:**: `null` if base is `null`; otherwise `Object.class`.


---


|  |  |  |
| --- | --- | --- |
| **Overview** \| **Package** \| **Class** \| **Use** \| **Tree** \| **Deprecated** \| **Index** \| **Help** | |  |
| **PREV CLASS**   **NEXT CLASS** | **FRAMES**    **NO FRAMES**     **All Classes** |  |
| SUMMARY: NESTED \| FIELD \| CONSTR \| METHOD | DETAIL: FIELD \| CONSTR \| METHOD |  |


---
